# Advances in the Prevention and Treatment of Obesity-Driven Effects in Breast Cancers

**DOI:** 10.3389/fonc.2022.820968

**Published:** 2022-06-22

**Authors:** Kuo Chen, Jin Zhang, Narasimha M. Beeraka, Chengyun Tang, Yulia V. Babayeva, Mikhail Y. Sinelnikov, Xinliang Zhang, Jiacheng Zhang, Junqi Liu, Igor V. Reshetov, Olga A. Sukocheva, Pengwei Lu, Ruitai Fan

**Affiliations:** ^1^ Cancer Center, The First Affiliated Hospital of Zhengzhou University, Zhengzhou, China; ^2^ Department of Human Anatomy, I. M. Sechenov First Moscow State Medical University of the Ministry of Health of the Russian Federation (Sechenov University), Moscow, Russia; ^3^ Center of Excellence in Molecular Biology and Regenerative Medicine (CEMR), Department of Biochemistry, JSS Academy of Higher Education and Research (JSS AHER), JSS Medical College, Mysuru, India; ^4^ Discipline of Health Sciences, College of Nursing and Health Sciences, Flinders University, Adelaide, SA, Australia

**Keywords:** breast cancer, obesity, oncogenic signaling, inflammation, estrogen, neoadjuvant therapy, preventive measures

## Abstract

Obesity and associated chronic inflammation were shown to facilitate breast cancer (BC) growth and metastasis. Leptin, adiponectin, estrogen, and several pro-inflammatory cytokines are involved in the development of obesity-driven BC through the activation of multiple oncogenic and pro-inflammatory pathways. The aim of this study was to assess the reported mechanisms of obesity-induced breast carcinogenesis and effectiveness of conventional and complementary BC therapies. We screened published original articles, reviews, and meta-analyses that addressed the involvement of obesity-related signaling mechanisms in BC development, BC treatment/prevention approaches, and posttreatment complications. PubMed, Medline, eMedicine, National Library of Medicine (NLM), and ReleMed databases were used to retrieve relevant studies using a set of keywords, including “obesity,” “oncogenic signaling pathways,” “inflammation,” “surgery,” “radiotherapy,” “conventional therapies,” and “diet.” Multiple studies indicated that effective BC treatment requires the involvement of diet- and exercise-based approaches in obese postmenopausal women. Furthermore, active lifestyle and diet-related interventions improved the patients’ overall quality of life and minimized adverse side effects after traditional BC treatment, including postsurgical lymphedema, post-chemo nausea, vomiting, and fatigue. Further investigation of beneficial effects of diet and physical activity may help improve obesity-linked cancer therapies.

## 1 Introduction

The current underlying mechanisms by which obesity can induce cancer include (a) substantial amounts of growth factors such as insulin and insulin-like growth factor 1 (IGF-1); (b) enhanced levels of sex steroid hormones, primarily estrogen, and other factors affecting metabolism; (c) changes in adipokines (leptin, adiponectin, and visfatin) that are typically involved in modulating the immune, tumor-regulatory mechanisms; and (d) low-grade inflammation and cytokine factors involved in fostering oxidative stress. Furthermore, recent evidence reported that obesity-driven changes in the intestinal flora microbiome promoted cancer incidence and progression (1–4).

In obese patients, body mass index (BMI) levels are extremely enhanced, while moderately increased BMI can improve overall survival and responses to therapy (4). The protective effect of moderately increased BMI is lost when BMI reaches the morbid obesity level, which is often referred to as “obesity paradox.” The effect of morbid obesity level on the response to cancer treatment remains poorly understood (5). This phenomenon was less examined in BC and some studies applied the inadequate use of BMI for overweight patients, missing the point that muscle mass could also contribute to the BMI (6). The higher adipose tissue mass may indicate energy stores that ensure a longer time in some patients during devastating effects of chemotherapy. Enhanced adipose stores may provide an energy reserve, which enables a longer survival time (7). This study aimed to evaluate controversial cancer and obesity-related concepts, noting that obesity-induced chronic inflammation may promote cancer, whereas a low BMI can also generate an immunodeficient state and confer the immune escape of cancer cells. Furthermore, once the cancer has been diagnosed and treated in obese individuals, the pro-inflammatory state may support the immune response against the tumor (7).

In premenopausal women, ovaries are the major source of estrogen. However, the adipose tissue is the significant source of estrogen in postmenopausal women (8). Previous studies have reported that there is an increasing risk of developing estrogen-dependent BC in obese postmenopausal women. A systemic chronic inflammatory state accompanies altered metabolism in obese individuals, evidenced by the rise in both inflammatory cells and inflammatory biochemical markers (9). Furthermore, an association between obesity and triple-negative breast cancers (TNBCs) has been demonstrated (5–7). The association between obesity, cardiovascular disease, and type 2 diabetes mellitus (10, 11) is well documented in the literature, but evidence linking obesity-triggered inflammation to cancer is scarce. Therefore, it is important to clarify whether obesity-induced inflammation plays a vital role in the pathogenesis of cancer and what are the associated mechanisms of this process.

A higher BMI is also associated with higher morbidity and mortality in BC patients (12). Abnormal adipocyte hypertrophy can lead to hypoxia and initiation of inflammatory responses. Inflammatory mediators such as interleukin 6 (IL-6) and tumor necrosis factor-α (TNF-α) are known activators of the nuclear factor (NF)-κB signaling pathway, the upstream regulator of aromatase expression in adipose stromal cells in the breasts (13). Aromatase is the key enzyme of estrogen synthesis, the growth-stimulating hormone for BC cells. Obesity and its involvement in the regulation of estrogen synthesis and pro-inflammatory conditions have been shown to influence BC recurrence, distant metastasis of TNBCs, and the overall survival of patients (14, 15). Despite significant advances in our understanding and management of patients with BC, the therapeutic focus can shift toward identifying the patients at higher risk of developing resistant BC in patients with high BMI and chronic low-level inflammation. The obesity-targeting therapies may facilitate novel ways to reduce the development and progression of BC.

In this review, we identified and analyzed 86 relevant keyword-linked papers, screened out of 256 research articles that reflect the impact of obesity on BC development, treatment/prevention approaches (16–18), and posttreatment pathologies. Additionally, we summarized the relationship between BC, obesity, inflammation, and estrogen signaling network. Our discussion provides new insight on obesity-linked prophylaxis and therapeutic management of estrogen-dependent BC in obese postmenopausal women. Our analysis is also focused on the role of obesity in modulating BC occurrence and immunotherapy efficiency (16–18) in obesity-driven TNBCs. We accent complementary BC treatment strategies including dietary practices and physical activity.

## 2 Literature Search and Data Collection

We conducted a systematic review using the indicated keywords and limited the search to the published meta-analyses and review articles that addressed the impact of obesity on BC development, treatment/prevention approaches, and posttreatment pathologies. PubMed, Medline, eMedicine, National Library of Medicine (NLM), and ReleMed were screened to retrieve practical meta-analysis studies using keywords/phrases as follows: “obesity,” “inflammation,” “surgery,” “conventional therapies,” and “diet.” A total of 86 research papers were considered suitable and included in the final review.

### 2.1 Study Selection (Inclusion/Exclusion Criteria)

The selected articles addressed the obesity paradox and potential roles of obesity and chronic inflammation in BC progression. Furthermore, articles that discussed BC surgery and other conventional therapeutic modalities were also included for the analysis of information related to specific recommendations on patient management. A total of 462 papers were identified using keywords “obesity,” “inflammation,” “multiple oncogenic pathways,” “breast cancer,” “surgery,” “conventional therapies,” and “diet.” During primary screening, the actuality, publication date, access to article text, and the content were considered, resulting in the selection of 74 research papers. After removal of duplications and full-text review, a total of 86 papers were considered as suitable for final review (Flowchart). Analysis of references contributed 23 additional articles.

## 3 Lifestyle Factors Driving the Incidence of Breast Cancer

According to the World Health Organization (WHO), obesity, electromagnetic pollution, environmental pollution, smoking, alcohol consumption, shift work factors, and usage of excessive antiperspirants and breast implants could be major significant factors conducive to BC development (19). For instance, body weight has been playing a prominent role in modulating inflammation, serum leptin, and estrogen and adiponectin levels subsequently associated with an increased postmenopausal BC risk (20). Furthermore, it has been delineated that obesity at the time of childhood and adolescence has an inverse relation with premenopausal incidence of BC without the influence of adult BMI (21–23). A mitigated BC risk has been observed upon a weight loss of 4.5 kg during the age period of 18–30 years in women in the premenopausal period (24).

Smoking is another lifestyle factor, which has a significant influence on the development of BC, as majority of carcinogens in cigar smoke could induce polymorphism in N-acetyltransferase-2, which in turn enhances the chances of BC development (25). Alcohol consumption can enhance mammary epithelial cell growth and induce enhancement in serum estradiol levels during premenopause conditions, which consequently provoke BC development in women (26). A few reports concluded that the risk of BC development is significantly higher with every additional 10 g of ethanol consumption on a daily basis (27–29). In case of shift-based work, mainly the women who have been working on night shifts are more prone to BC development when compared to day shift workers (30–32). Antiperspirant usage is another lifestyle-driven risk factor in women that can drive the incidence of BC, as majority of deodorants are composed of parabens, which have significant estrogenic properties (33–35). In addition, aesthetic surgeries using breast implantations (for example, silicone breast implants) for a better breast augmentation could be another risk factor that can induce malignancy in breast tissue, which yet requires substantial research studies (36–39).

## 4 Obesity-Linked Effects on Breast Cancer Incidence, Treatment, and Prognosis

### 4.1 Obesity Is Associated With Activation of Multiple Oncogenic Pathways in Breast Cancers

The obesity-driven BC pathology was associated with the activation of several oncogenic pathways, including leptin signaling network and oxidative stress mechanisms. BC is marked by increased reactive oxygen species (ROS) production in epithelial mammary cells (40), although the chronically increased leptin could mitigate ROS levels in MCF-7 cells (41). Leptin can regulate metabolic reprogramming during activated cell growth, control autophagy, and inhibit BC cell apoptosis (42, 43). Leptin can modulate both tumor–stromal interactions (44) and activity of M2 type macrophages *via* generation of IL-18 and IL-8 that foster BC growth and metastasis (45). Leptin was also shown to enhance BC stemness *via* enhanced expression of stemness/epithelial–mesenchymal transition (EMT)-related genes (46). Activated leptin receptors promote several signaling cascades through the canonical pathways, such as Janus kinase 2 (JAK2)/signal transducer and activator of transcription (STAT) proteins, mitogen-activated protein kinase (MAPK/ERK1/2), phosphoinositide 3-kinase (PI3K)/V-akt murine thymoma viral oncogene homolog (AKT), and noncanonical signaling *via* protein kinase C (PKC), c-Jun N-terminal kinase (JNK), p38 MAPK, and AMP-activated protein kinase (AMPK) (20–25). BC progression and proliferation are regulated by leptin-mediated JAK2/STAT3 signaling in patients with obesity-like conditions (47). Leptin also regulates the cell cycle and enhances the cyclin D1 expression *via* the STAT3 network (48). BC proliferation can be triggered by enhanced hTERT activity and expression that are under the control of STAT3 phosphorylation (49). STAT3 signaling influences EMT and foster BC invasion and migration (50). TNBC chemoresistance and cancer stem cell proliferation were shown to be controlled by leptin-induced STAT3 (51, 52). Leptin can modulate BC proliferation *via* MAPK/ERK1/2 signaling, the pathway that was linked to estrogen receptor alpha (ERα) and aromatase activities (53).

In obese patients, leptin signaling correlated with the increased risk of BC (54, 55) *via* activation of prosurvival effectors, including PI3K/AKT, and activation of EMT (56, 57) (**Figure 2**). PI3K/AKT/SREBP2 signaling axis can enhance the expression of acetyl-CoA acetyltransferase 2 (ACAT2) and promote BC proliferation and migration (58). The signaling cascade that is triggered by PI3K/AKT/mammalian target of rapamycin (mTOR) pathway was shown to enhance levels of insulin, which can promote mitogenesis and growth of BCs, which often express type A insulin receptors (59–61). Insulin can enhance the production of IGF-1 and mitigate IGF-1-binding protein activation, consequently promoting tumor growth (62–65) (**Figure 2**). Higher IGF-1 activity and IGF-1 receptor expression were reported in different types of BCs, including TNBCs (**Figure 2**) (66). Insulin and estrogen signaling can coordinate therapy responses in many BCs. Natural estrogen 17β-estradiol binds ERα and promotes cancer growth in ER+ BCs (15). Previous reports indicated that gut microflora can affect IGF-1 and estrogen responses, estrogen-regulated metabolism, and estradiol production in obese postmenopausal BC patients (67). For instance, 17H-hydroxysteroid dehydrogenase 1 gene (17HSD1), which is located near the BReast CAncer susceptibility gene 1 (BRCA1) in the human genome, effectively controls the conversion of estrone to estradiol and stimulates BC growth (68, 69). Whether the activation of estrogen signaling axis is facilitated or inhibited by a low-profile inflammation and leptin-activated network in BC patients remains unclear.

### 4.2 Obesity and Metabolic Transformation as Triggers of Inflammation and Oncogenesis

**Figure 1:**
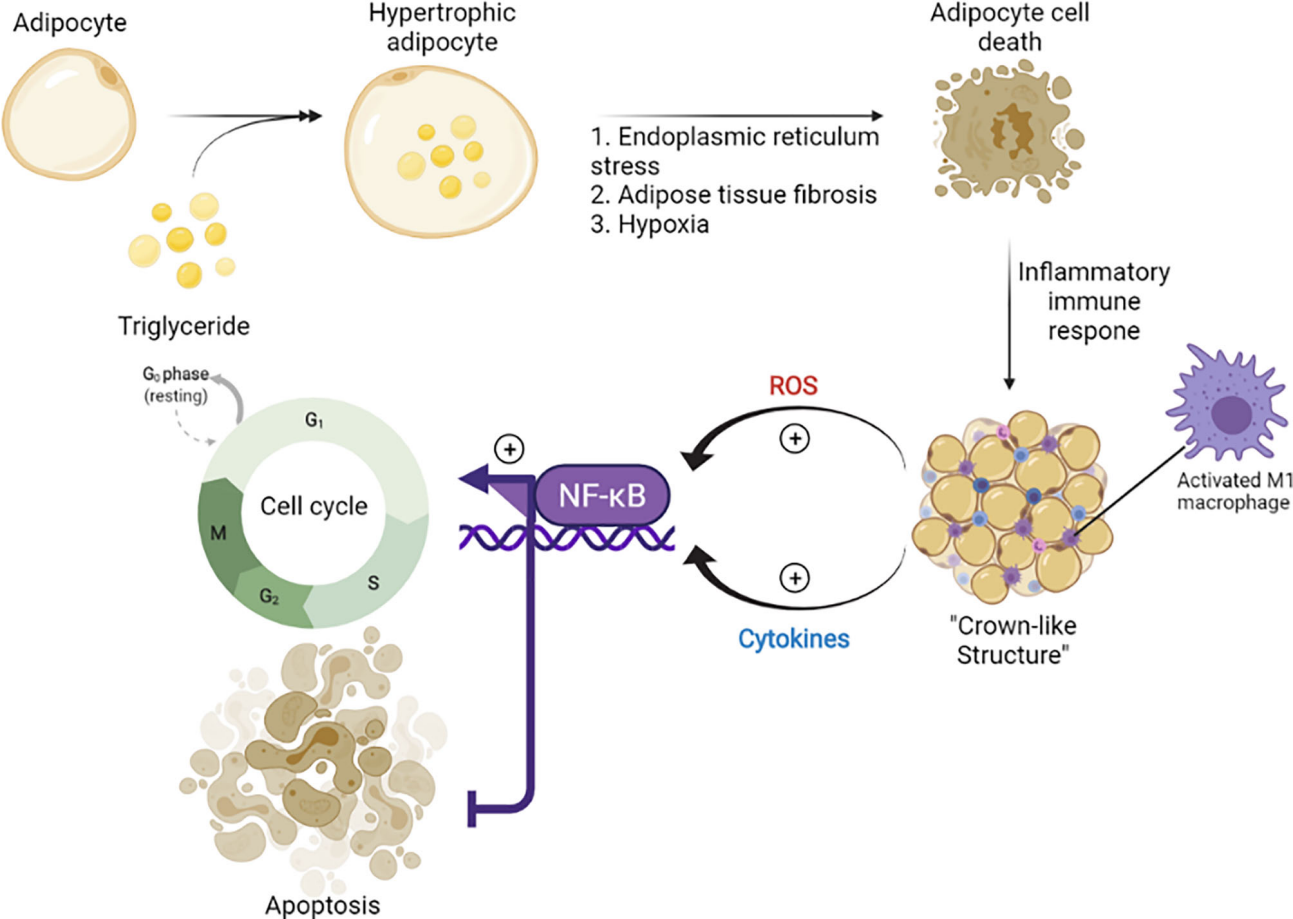
The role of obesity in the stimulation of carcinogenesis (71). Chronic low level of inflammation was reported in obese individuals and associated with the inflammatory immune responses initiated by the death of adipocytes. During this process, M1 macrophages are accumulated and form a crown-like structure, which provokes cell cycle progression in adipose stromal cells of the breast *via* secretion of inflammatory mediators (TNF-α, IL-6, IL-11) and activation of NF-κB. tumor necrosis factor-alpha, TNF-a; interleukin 6, IL-6; interleukin 11, IL-11; nuclear factor kappa-light-chain-enhancer of activated B cells, NF-kB.

Epidemiological studies indicated the role of obesity as a driving force of BC. The excess calories stored as triglycerides in adipocytes can lead to endoplasmic reticulum stress, adipose tissue fibrosis, and hypoxia, which result in adipocyte cell death and initiation of inflammatory immune responses (70). Especially in obese individuals, chronic inflammation is observed, marked by the formation of a distinct histological structure (“crown-like structures”) (**Figure 1**), associated with the infiltration and accumulation of activated M1 macrophages in adipose tissue (72). The M1 macrophages (73) are involved in the promotion of inflammation, generation of ROS, and release of pro-inflammatory cytokines (IL-6 and TNF-α) (74). TNF-α, IL-6, IL-11, leukemia inhibitory factor, and prostaglandin-E2 have been shown to activate NF-κB signaling pathway and increase the expression of aromatase in breast adipose stromal cells (13). The effect led to the suppression of apoptosis and promotion of cell cycle progression (75). The roles of mitochondria and ROS are well defined in obesity-mediated BC progression (76). The excessive energy stores in obese patients can result in mitochondrial dysfunction (77) that is associated with carcinogenic transformation and metastasis (78).

Procarcinogenic effects of estrogen were registered *in vivo* using large doses of natural and synthetic estrogens in experimental animals (79). The adipocytes synthesize substantial levels of estrogen, which can also cause ER+ BC progression (80). However, estrogen, as an important steroid hormone, is essential for normal functioning of endocrine-responsive tissues. Estrogen is used as an oral contraceptive and prescribed as treatment for osteoporosis, cardiovascular diseases, atrophic vaginitis, hypertension, and stroke in postmenopausal women. Estrogen can increase the levels of apolipoproteins A-I and high-density lipoproteins (HDLs) (“good cholesterol”), while the level of low-density lipoproteins (LDLs) (“bad cholesterol”) can be decreased by this steroid hormone (81).

However, the precise biological mechanism of estrogen-induced cancer progression and spreading remains unclear. One of the cancer-promoting mechanisms is associated with transactivation of growth factor receptors. Epidermal growth factor receptor (EGFR) transactivation by estrogen is an important signaling mechanism in the regulation of cancer cell growth (82). Several hypotheses were also reported that described ER signaling as a trigger for precancer cell transformation into malignant cells and the shift of dormant cells from G0 to G1-S active phase (**Figure 1**). This process was referred to as “estrogenic recruitment.” During this activation, the synthesis of growth factors could promote tumor growth and pro-oncogenic signaling, increase the formation of free radicals, inhibit apoptosis in transformed cells, and stimulate the synthesis of cathepsin D and plasminogen activator factor (PAF) required for BC progression (79).

It has been observed that 5′-AMPK, liver kinase B1 (LKB1), and the metabolic sensors are crucial modulators of ATP synthesis and energy utilization (83). The LKB1/AMPK axis inhibits nuclear entry of tCREB-coactivator (cAMP response element-binding protein) and CRTC2 (CREB-regulated transcription cofactor 2). These factors ultimately lead to the inhibition of aromatase expression in the isolated adipose stromal cells (84) and may significantly impact BC progression. This mechanism may be explored and utilized for therapeutic interventions in obese BC patients.

### 4.3 Obesity and ER+ Breast Cancer in Postmenopausal Women

The positive association between obesity and the risk of ER+ BC in postmenopausal woman has been reported (85). Interestingly, a higher BMI in young women (at the age of 18 years) correlated with the lowest BC rate before and after menopause (65). The weight gain after 18 years of age in women was not related to the incidence of BC before menopause, while it was positively linked to the higher risk of BC after menopause (86). The mitigation of body fat levels was positively associated with decreased circulating estrogen and inversely associated with the risk of estrogen-dependent BC in postmenopausal women (87, 88). Several studies demonstrated that weight loss is associated with the decrease in serum C-reactive protein (CRP) (89–91), IL-6 (91), and serum amyloid A (92) in postmenopausal women. Since most (nearly 80%) BCs in postmenopausal women are estrogen-dependent, prospective therapeutic interventions should focus on reducing chronic inflammation, fat-linked estrogen production, and weight loss. Another study by Tchernof et al. (93) suggested calorie restriction and the intake of 1,200 kcal a day for an average of 13.9 months to reduce the level of circulating estrogens. This approach resulted in an average weight loss of 14.5 kg and serum estradiol reduction of 21.7 pg/ml (93).

The high level of BMI significantly impacts BC prognosis. A higher production of peripheral estrogen in adipose tissues correlated with the higher BMI in postmenopausal patients. A minimal level of sex hormone-binding globulin was observed in patients with a high BMI and could be responsible for poor BC prognosis. The substantial increase in aromatase activity and estrogen synthesis was also linked to uncontrolled proliferation of BC cells (94, 95). Therefore, an elevated BMI may negatively impact BC patients receiving aromatase inhibitors (96). However, older BC patients with higher BMI may benefit and better withstand chemotherapy and the associated adverse toxicity (97). It is unclear whether all BC patients with a high BMI acquire higher insulin, IGF, and steroid hormone levels that could provoke a substantial mitogenic activity (98). Notably, cytokines generated in the adipose tissue of obese individuals may enhance BC progression through the upregulation of stem cell signaling, metastasis, and angiogenesis (99, 100). Several studies reported that premenopausal and perimenopausal BC patients with a higher BMI (estimated as obesity or overweight) indicated poorer prognosis regardless of the tumor subtype (101–104). A higher BMI ≥25.8  kg/m^2^ also resulted in higher mortality in premenopausal patients (71, 83).

### 4.4 Clinical Reports of Therapies Targeting Oncogenic Pathways in Breast Cancers

BC can be categorized into 4 subtypes according to molecular markers and mammary epithelial biology (105). The most common molecular markers used for disease prognosis and methods of treatment (106) are presented in **Table 1**. BCs expressing hormone receptors, i.e., ER (ER+) or progesterone receptor (PR+), are classified as luminal A and luminal B BC types (107). Erb-B2 receptor tyrosine kinase 2 (ERBB2+/HER2+) cancers are characterized by human epidermal growth factor receptor 2 (HER2) gene amplification (109). Even though estrogen has been proven as a potent mitogen in ER+ BCs, it has been found that patients with this type of malignancy have a relatively better prognosis (111). **Table 2** presents epidemiological BC data [2014–2018 described by the National Institutes of Health (NIH)] and summarizes information about the four BC subtypes.

**Table 1:** Molecular subtypes of BC.

BC molecular subtypes	Definition	Pathogenesis	Subtype-specific management
HR+/ERBB2- (“Luminal A”)	Tumor cells show higher levels in the expression of estrogen/progesterone receptor proteins and low level of ERBB2 proteins (107)	Estrogen receptor-α activates oncogenic growth pathway (104)	Endocrine therapy (all) Chemotherapy (some) (105)
HR-/ERBB2- (“Triple Negative”) (“Basal-like”) (108)	Tumor cells do not meet any pathologic criteria for positivity of estrogen/progesterone receptor or ERBB2 (109)	BRCA1 mutation	Chemotherapy therapy (all)
HR+/ERBB2+ (“Luminal B”)	Tumors strongly express ERBB2 protein or show ERBB2 gene amplification and are positive for estrogen/progesterone receptor proteins (107)	ERBB2 that encodes ERBB2 receptor tyrosine kinase from the epidermal growth factor receptor (EGFR) family is overexpressed. Estrogen receptor α activates oncogenic growth pathway	Chemotherapy+ERBB2-targeted therapy (all) Endocrine therapy (all)
HR-/ERBB2+ (“HER2-enriched”)	Tumors strongly express ERBB2 protein or show ERBB2 gene amplification and are negative for estrogen/progesterone receptor proteins (110)	ERBB2 that encodes ERBB2 receptor tyrosine kinase from the epidermal growth factor receptor family is overexpressed	Chemotherapy+ERBB2-targeted therapy (all)

BC, Breast Cancer; HR, hormone receptor; ERBB2, Erb-B2 receptor tyrosine kinase 2; BRCA1, BReast CAncer gene 1.

**Table 2:** Statistics based on 2014–2018 cases according to the National Institutes of Health (112).

		HR+/ERBB2-(“Luminal A”)	HR-/ERBB2-(“Triple Negative”)	HR+/ERBB2+(“Luminal B”)	HR-/ERBB2+(“HER2-enriched”)	Unknown
Age-adjusted rate of new cases per 100,000 by subtype		88.1	13.1	13.4	5.5	8.8
Age-adjusted rate of new cases per 100,000 by race	White	92.3	12.1	13.4	5.2	8.5
	Black	71.9	22.8	13.5	6.6	9.6
	AI/AN	54.4	7.6	8.6	4.1	6.7
	API	70.4	8.6	12.6	5.9	7.0
	Hispanic	64.3	11.0	11.2	4.9	8.5
Percent of cases		68%	10%	10%	4%	7%
5-YRSP by subtype		94.3%	76.9%	90.5%	84.0%	76.1%
5-YRSP by stage	Localized	100.0%	91.2%	98.9%	96.7%	95.8%
	Regional	89.9%	65.4%	89.4%	82.0%	77.1%
	Distant	30.6%	12.2%	44.7%	37.9%	16.4%

HR, hormone receptor; ERBB2 or HER2, human epidermal growth factor receptor 2; 5-YRSP, 5-year relative survival percent; AI/AN, American Indian/Alaska Native; API, Asian or Pacific Islander.

**Figure 2:**
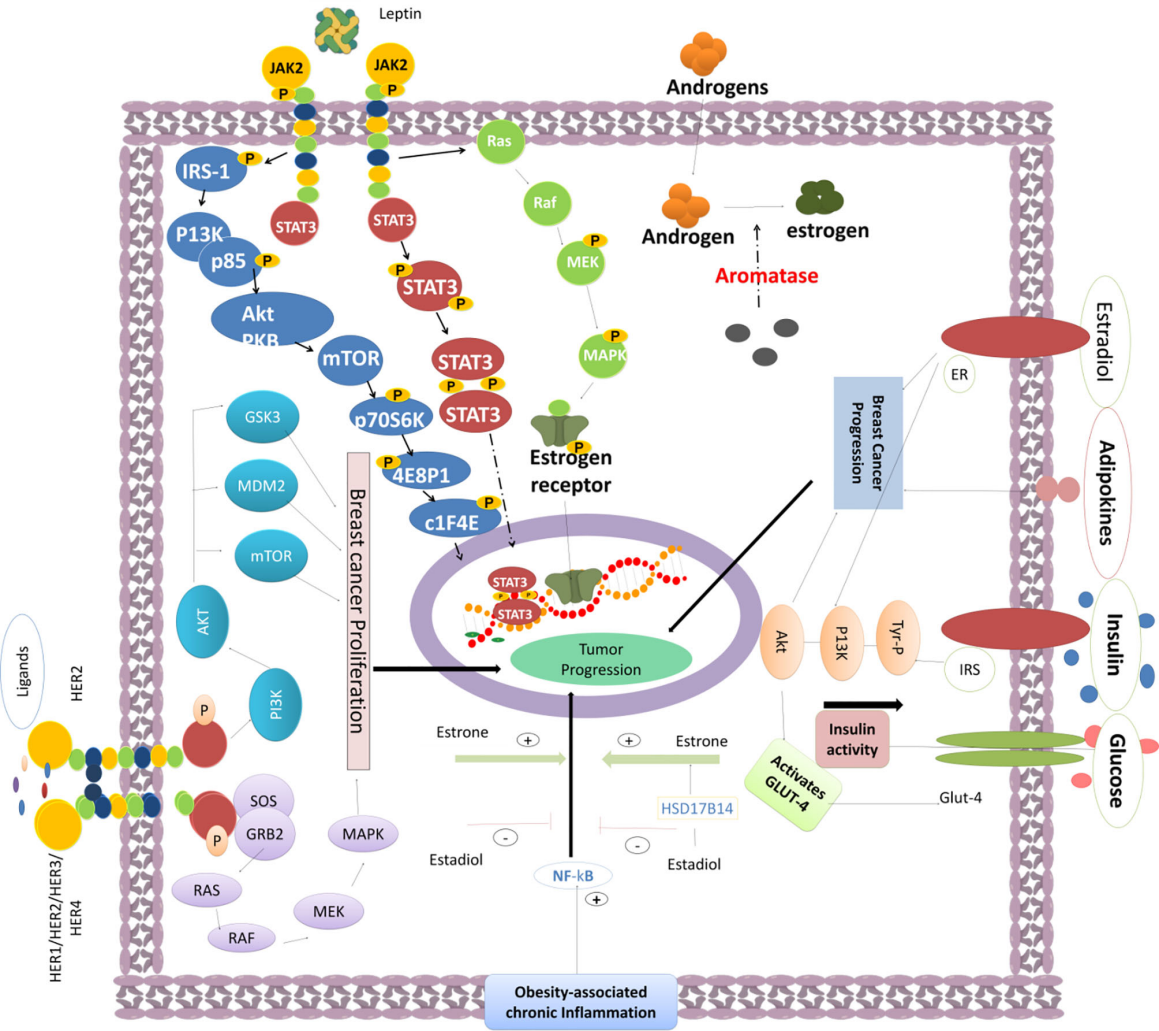
The activity of various oncogenic pathways triggers BC epithelial cell proliferation and tumor progression. Obesity-induced chronic inflammation can promote the NF-κB signaling. Fat cell-produced estrone can enhance the activation of NF-κB and promote BC progression. Leptin can modulate the PI3K/AKT signaling through IRS-1, enhance mTOR activity, trigger 4EBP1 and elF4E phosphorylation, and mediate the STAT3-promoted gene transcription required for cancer cell growth. Furthermore, the JAK-STAT and MAPK pathways are enhanced by the leptin-induced JAK2 signaling. MEK phosphorylation strongly promotes gene expression for efficient tumor progression. Aromatase activity promotes the androgen to estrogen conversion required for tumor progression. Other signaling effectors such as insulin, estradiol, adipokines, and high glucose can also enhance BC progression. Insulin can promote IRS and Tyr-P signaling and induce the activation of PI3K/AKT *via* stimulation of GLUT-4, which consequently enhances the glucose uptake into BC cells. Several HER1/HER2/HER3/HER4 ligands can enhance the activity of HER2, promote the tyrosine kinase domain phosphorylation, and induce BC progression through the signaling cascade mediated through the PI3K/AKT/mTOR and RAS-RAF-MEK-MAPK pathways to foster cell growth and proliferation.

Hormone replacement therapy (HRT) produced a favorable clinical outcome against luminal A ER+ tumors (111). HRT is considered as the best option for patients with highly endocrine-responsive tumors, while cell cycle targeting (cytotoxic) chemotherapy is often used with non-endocrine-responsive tumors. A combinatorial regimen of cytotoxic drugs (113) along with endocrine therapy is defined according to the tumor size, grade, presence of peritumoral vascular invasion, and nodal status. Ki67 is one of the crucial parameters that is used during the decision about chemotherapy approach. A combinatorial regimen with HRT is often prescribed for the treatment of obese BC patients with Luminal-A molecular subtypes (114). The administration of trastuzumab can be beneficial in patients with HER2+ BCs with more than 10% invasive tumor cells or showing HER2 gene amplification (115). Additionally, conventional TNBC therapy involves combined HRT (116) and immunotherapy agents (117). However, application of these therapeutic agents was associated with the development of multidrug resistance (MDR) in obese/overweight patients (117). Several other effective chemotherapeutic drugs, including antitubulin agents, platinum agents, EGFR inhibitors, antiangiogenic agents, androgen receptor (AR) antagonists, histone deacetylase (HDAC) inhibitors, PI3K/AKT/mTOR pathway inhibitors, MAPK/MEK modulators, and checkpoint kinase 1 (Chk-1) inhibitors, were shown to target oncogenic signaling pathways (**Figure 2**) (118–121). Neoadjuvant chemotherapy with these agents (122–124) is a multicomponent therapeutic regimen used in advanced BC (125, 126).

#### 4.4.1 Obesity in Patients With Triple-Negative Breast Cancers and the Effectiveness of Chemotherapy

The first generation of chemotherapy, for example, cyclophosphamide, methotrexate, 5-fluorouracil (5-FU), can induce 35% reduction in BC mortality when compared to adjuvant chemotherapy (127). However, the adverse effects of chemotherapy (128–133), such as alopecia, fatigue, nausea, vomiting, myelosuppression, acute leukemia, cardiac toxicity, and neuropathy (134), should also be considered. Adjuvant systemic chemotherapy was shown to induce less adverse side effects while delivering sufficiently decreased metastasis and BC mortality rate (135). However, the development of resistance to chemotherapy was observed in nearly 40% of BC patients during the first 5 years of treatment. TNBCs belong to the advanced type of malignancies that demonstrate lower rates of chemotherapy response and higher rates of resistance. TNBC patients are diagnosed with a relatively larger tumor size and higher T stage and tumor grade. TNBCs are often detected in obese patients. Previous reports demonstrated that a substantial portion of obese/overweight patients is diagnosed with TNBC (136). Another retrospective study reported that the incidence of TNBC in obese individuals is significantly higher than that in non-obese TNBC patients (137).

HR-negative (HR-) BCs (including TNBC) exhibit a higher disease recurrence rate compared to HR-positive (HR+) BCs (138). AR antagonists (such as nonsteroidal anti-androgen bicalutamides) demonstrated good clinical efficacy in Phase II single-arm trials in TNBC patients harboring AR-positive tumors (139). However, the clinical efficacy of AR antagonists is still debatable and/or controversial. The modest pharmacological activity of bicalutamides may be associated with the indolent nature of the luminal disease.

The recurrence rate could be decreased by the application of adjuvant chemotherapy prior to surgical intervention with good clinical outcomes if administered before (neoadjuvant) or after (adjuvant) surgery (140, 141). Breast-conserving surgery is commonly followed by the administration of systemic therapeutic regimens to enhance clinical responses (neoadjuvant therapy). However, in obese TNBC patients, the risk of relapse is high (120–123). The clinical trial design should target overweight/obese patients as a separate cohort and test novel therapeutic agents mainly against TNBCs in obese individuals (142). TNBCs often express EGFR, and preclinical research demonstrated that bilateral BCs could be treated using anti-EGF therapeutic agents, including EGFR inhibitors (141, 143). The combined EGFR inhibitor therapy (cetuximab with platinum) was evaluated for its effectiveness in clinical trials (144). However, more studies are warranted to address chemotherapy responses in obese BC cohorts.

#### 4.4.2 Histone Deacetylase Inhibitors

**Figure 3:**
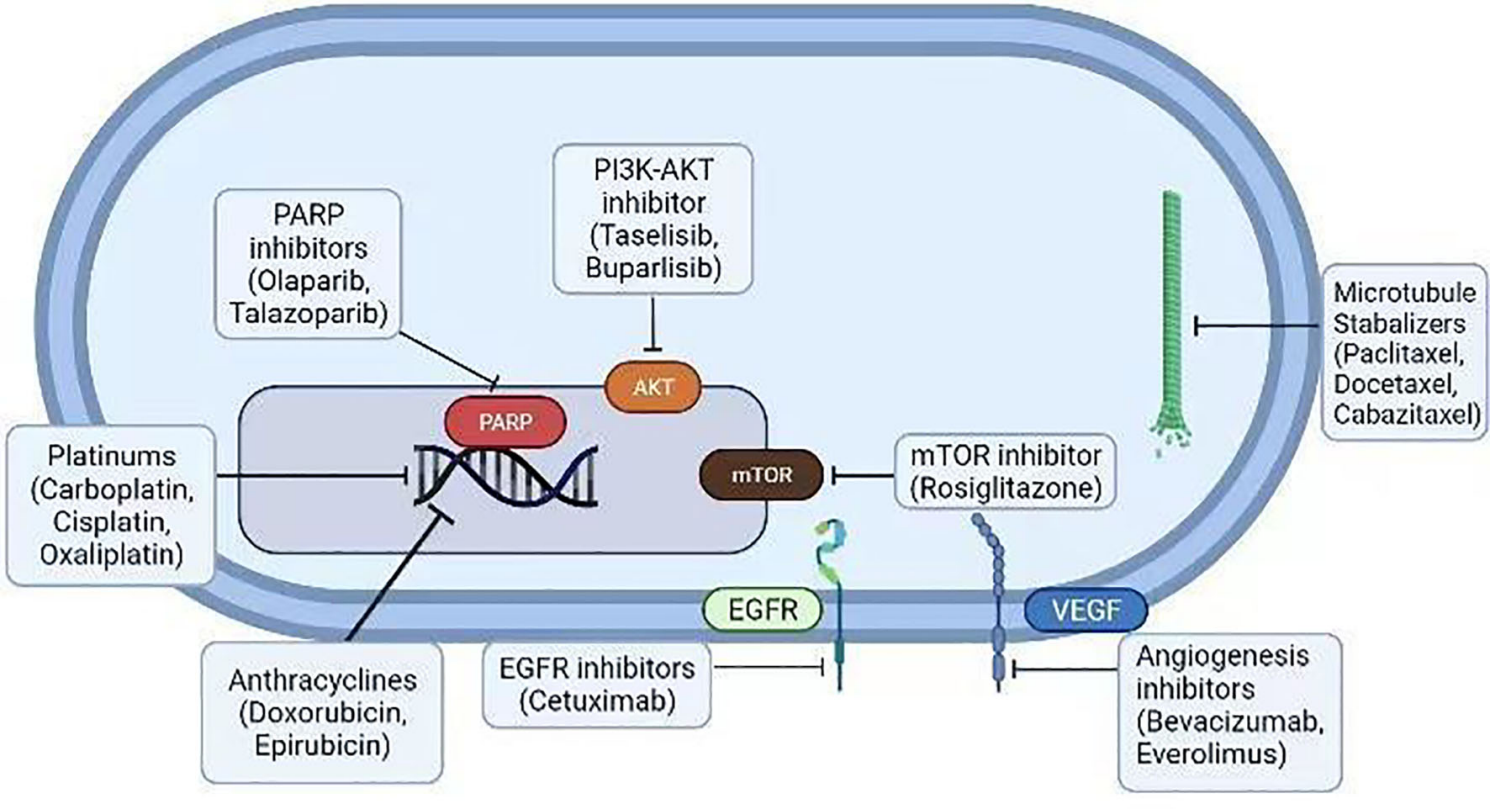
Various conventional therapeutic agents (platinum drugs, anthracyclines, EGFR blockers, mTOR blockers, angiogenesis inhibitors, microtubule stabilizers, and PARP and PI3K-Akt inhibitors) were examined against the BCs. Many of them are FDA-approved drugs for BC therapy.

Histone deacetylase (HDAC) inhibitors were used to target epigenetic regulation by HDAC, the enzyme involved in chromatin modeling and gene transcription in TNBCs. HDAC inhibitors modulate the acetylation and deacetylation of core histones, thereby reprogramming the expression of various genes involved in controlling cell proliferation, survival, metastasis, and angiogenesis (145, 146). HDAC inhibitors were tested against TNBC in preclinical models. For instance, the combination of vorinostat with neoadjuvant therapeutic regimens of carboplatin and nab-paclitaxel has not delivered improved pathological complete response (pCR) rates in TNBC patients during randomized Phase II trials. HDAC inhibitors provoked genome-wide effects by enhancing the reexpression of ER or BRCA1/2, which is accompanied by the silencing of other tumor-suppressive genes (147). Hence, the identification of new classes of HDAC inhibitors to control the functions of tumor-promoting genesis in obese TNBC patients is warranted (**Figure 3**).

MAPK kinase/MEK pathway activation in the TNBC is mediated by the upstream receptor tyrosine kinases (RTKs) (148). Impairment of MEK could induce compensatory mechanisms and trigger a variety of RTKs in TNBCs (148). Molecular signaling that involves PI3K/AKT activation was reported in several malignancies including BRCA-associated BCs in obese patients (149, 150). The assessment of clinical efficacy of MEK inhibitors (for example, GSK1120212 or trametinib) and PI3K/AKT inhibitors against TNBC and other devastating malignancies is currently being addressed. Several trials aim to characterize the antitumor effects of these combinatorial regimens against both basal-like BCs and mesenchymal subtypes of TNBC (151, 152).

Immune Chk inhibitors are reported to be highly efficient against TNBC harboring p53 mutations (153). Cancer cells require Chk-1 to modulate the cell cycle. Previous studies used p-53-deficient mouse models of BC and showed the induction of apoptosis upon Chk-1 inhibition (153, 154). Immune modulation with Chk-1 inhibitors is a promising BC treatment strategy in obese individuals with aggressive cancer biology. Immunotherapy agents (blocking or activating specific targets), including PD-1 or CTLA-4 blockers (155), were approved for antitumor evaluation in clinical studies. The immunotherapy strategies that target tumor-infiltrating lymphocytes warrant testing in TNBC patients (155).

#### 4.4.3 Poly ADP-Ribose Polymerase Inhibitors Against Triple-Negative Breast Cancer

The poly ADP-ribose polymerase (PARP) family includes enzymes responsible for DNA repair. DNA damage activates nuclear PARPs that are required for DNA repair in BC cells (156). PARP activation contributes to the recruitment of base excision repair (BER) to the site of DNA damage and fosters chromatic relaxation to induce DNA repair (157–159). Hence, PARP inhibitors could induce synthetic lethality when administered in combination to target TNBCs in obese individuals. Successful TNBC cell survival and proliferation depend on DNA repair pathways and other homologous recombination repair (HRR) mechanisms. Olaparib is a PARP inhibitor primarily examined as a choice of chemotherapy against germline BRCA mutated (gBRCAm) HER2-negative (HER2-) BCs (142). This drug invoked radiological responses in 60% of patients compared to the 29% of the group receiving chemotherapy alone. Furthermore, TNBC progression was delayed from 4.2 to 7.0 months by the PARP inhibitor treatment. The clinical efficacy of PARP inhibitor combinatorial regimen (carboplatin with veliparib to Adriamycin^®^) was ascertained in comparison to cyclophosphamide (AC)-paclitaxel or AC-paclitaxel with carboplatin (160). The observed effects supported the addition of carboplatin that improved the clinical benefit of veliparib, although further testing is required (161). Synthetic lethality was a more profound clinical effect observed during PARP inhibition. PARP1 enzymatic inhibition could foster the formation of unrepaired single-strand DNA breaks, which consequently induce cell death in cancer cells defective in homologous recombination DNA repair (HRR) mechanisms. Thus, the molecular signaling of PARP could be impaired by targeted inhibition, producing potent cytotoxic effects in BC cells. Accordingly, the administration of olaparib, a strong PARP-trapping agent (162, 163), was evaluated in combination with platinum drugs in OlympiA PARTNER neoadjuvant clinical study [ClinicalTrials.gov] (142). Talazoparib is another PARP inhibitor that exhibits PARP-trapping activity and catalytic efficacy. The agent produced a significant clinical response when used as neoadjuvant monotherapy in gBRCAm patients (164). In another clinical Phase II study, patients who were diagnosed with gBRCAm, TNBC, and HR+/HER2- were administered PARP inhibitor monotherapy for 6 months prior to surgical intervention. The treatment resulted in a 59% rate of residual cancer burden (RCB)-0/I (142). Considering the success of these regimens, the approach should be examined for clinical outcomes in obese/overweight BC patients with different BC molecular subtypes.

#### 4.4.4 Microtubule Stabilizers Against Triple-Negative Breast Cancer

Mammalian‐enabled (MENA) protein is an invasive form of microtubule protein that promotes BC cell proliferation and metastasis. MENA is an Ena/VASP family protein and is the most common elongation factor involved in fostering the cancer cell division including BC (159). To combat BC and improve clinical outcomes, microtubule-targeting therapy in obese patients with TNBC warrants considerations and testing (159). Taxane derivatives, including paclitaxel, docetaxel, and cabazitaxel, are potent tubulin-polymerizing agents that induce microtubule stabilization. They are more effective against TNBCs than ER+/PR+ BCs (165, 166). For instance, obese patients with TNBCs who have been receiving uncapped doses of taxane derivatives as neoadjuvant chemotherapy demonstrated higher pCR followed by good progression-free survival (PFS) (167).

The administration of paclitaxel for 8 weeks, as neoadjuvant after 4 treatment cycles of 5-FU, epirubicin, and cyclophosphamide, was accompanied by substantial enhancement in the clinical outcomes in TNBC patients (165). Ixabepilone is another microtubule stabilizer that is actively used for the treatment of TNBC patients (168, 169). However, the administration of this drug could be bypassed by chemoresistance mechanisms exerted by cancer cells. Therefore, ixabepilone is not as efficient as other anthracyclines and taxanes. Metastatic TNBC, treated with taxane/anthracycline, was shown to develop resistance, although combinatorial regimen with ixabepilone and capecitabine (a prodrug of 5-FU) delivered improved PFS compared to capecitabine alone (169). This combinatorial regimen could be a preferred therapeutic option for BC patients exhibiting cisplatin chemoresistance. Taxane-based chemotherapy is the recommended adjuvant regimen and one of the standard of care for patients with high-risk/high-grade/node-positive BCs, including TNBCs, HER2+, and HR+/HER2- cancers (170). Several studies and meta-analyses reported that the postsurgical anthracycline–taxane treatment was associated with enhanced relapse-free survival and better overall survival compared to the monotherapy of anthracycline alone (171, 172). The GeparTrio clinical study showed that patients who received 8 cycles of neoadjuvant TAC (docetaxel, doxorubicin, cyclophosphamide) combinatorial therapy displayed 37% of pCR rate in a TNBC cohort (173). However, the choice and schedule of soluble taxane derivatives remain to be assessed for clinical efficacy against TNBCs (166, 174). Albumin-bound paclitaxel (nab-paclitaxel) exhibited antitumor efficacy as neoadjuvant treatment in BC patients. The GeparSepto trial reported 38% increases in pCR rates with sequential combinatorial regimen (epirubicin, cyclophosphamide, and nab-paclitaxel) compared to the pCR rate displayed by the soluble paclitaxel alone given every week continuously for 12 weeks (175). Nab-paclitaxel was reported to induce lower anaphylaxis and hypersensitivity rates. ETNA clinical trials have reported better pCR with nab-paclitaxel (22.5%) compared to that with paclitaxel alone (pCR rate was 18.6%). However, the clinical use of taxane is limited due to the hypersensitivity observed in TNBC patients.

#### 4.4.5 Anthracyclines Against Triple-Negative Breast Cancers

Anthracycline derivatives are one of the significant chemotherapies used to treat TNBCs and other BCs by targeting DNA topoisomerase II (176). Doxorubicin and epirubicin are preferred neoadjuvant and adjuvant therapeutic agents in obese individuals with BCs. TNBCs exhibit chemosensitivity to anthracycline derivatives, although their clinical efficacies are controversial (177, 178). A retrospective study reported a 22% pCR rate with an epirubicin-containing regimen in TNBC patients, which was an increased rate compared to that in non-TNBC patients who exhibited an 11% pCR rate with the same regimen (177). TNBC patients demonstrated substantially improved clinical responses to doxorubicin and cyclophosphamide therapy compared to non-TNBC patients (178). Furthermore, a specific clinical benefit was observed with anthracycline and taxane combinations in node-positive TNBCs, although the efficacy of anthracyclines was not confirmed (179).

The WSG AM-01 trial indicated a clinical benefit of adjuvant anthracycline therapeutic modality in TNBC patients. The study compared a group of patients who received dose-dense conventional chemotherapy with another group that received a rapidly cycled high-dose regimen. The results indicated a 5-year event-free survival (EFS) rate equal to 71% in TNBC patients compared to 26% EFS for those who received the conventional dose-dense chemotherapy (180). However, the clinical efficacy of anthracycline-based chemotherapy remains controversial despite high response rates to anthracyclines alone as neoadjuvant treatment (181). The main reason for this controversy may be explained by the heterogeneous nature of TNBCs. Moreover, it is still unclear in terms of anthracycline sensitivity whether BRCA1-associated TNBC is functionally similar to sporadic disease (180, 182).

#### 4.4.6 Platinum Derivatives Against Triple-Negative Breast Cancer

Platinum-bound agents, including carboplatin, cisplatin, and oxaliplatin, can induce DNA double-strand breaks (DSBs). The agents facilitate DNA adduct formation, which consequently blocks DNA replication (183, 184). Furthermore, the platinum drugs are reported to be active against TNBC subtypes in obese individuals. The platinum-based drugs were shown to be highly effective regimens in obese individuals with BCs (185). The platinum-containing regimens induced a modest PFS improvement in metastatic TNBC but little to no effect on PFS or overall survival in non-metastatic BCs (185). Carboplatin was reported to be more effective in unselected metastatic TNBC when compared to docetaxel in first-line treatment of metastatic TNBC cases as reported in TNBC trials (186). Platinum derivatives could be considered as effective chemotherapeutic agents in patients with metastatic BCs. Clinical responses were observed in gBRCAm carriers but not in the epigenetic-driven BRCA carriers with TNBCs. A retrospective study of a large cohort of metastatic TNBC indicated the clinical efficacy of platinum-based drugs as first-line therapy with improved PFS compared to the patients without platinum therapy (187). The GeparSixto trial tested the effect of carboplatin that was added to paclitaxel and liposomal doxorubicin on a weekly basis for a continuous 18 weeks (preoperative period) in HR- BCs (188). In case of TNBC, the pCR rate was higher (57%) and patients exhibited a superior recurrence-free survival with carboplatin addition (189, 190). Thus, the addition of carboplatin to neoadjuvant therapy significantly enhanced pCR rates, but the survival-related data are currently unequivocal.

Cyclophosphamide is another anticancer drug used in TNBC therapy. This therapeutic molecule regulates alkylation and can induce cytotoxic effects by promoting the formation of DSB-mediated DNA interstrand cross-links. The GeparSixto study described that carboplatin and high-dose cyclophosphamide may be replaced by the combinatorial regimen of taxanes and anthracyclines and displayed similar pCR rates of 48.3% in intense dose-dense epirubicin, paclitaxel, and cyclophosphamide (iddEPC) arm and 48% in non-pegylated liposomal doxorubicin + carboplatin in TNBC (PMCb) arm (191). However, the administration of carboplatin has not been referred to as the standard of care (neoadjuvant therapy) against TNBC. The usage of this agent is still constrained by several adverse effects (neutropenia, anemia, and thrombocytopenia). It is essential to carefully define the patient subgroup that may benefit from this treatment approach and have a lower number of adverse complications. Moreover, in patients who were prescribed neoadjuvant therapy with platinum derivatives, the absence of BRCA is reported to be a good predictive biomarker (192).

#### 4.4.7 Antiangiogenesis Agents Against Triple-Negative Breast Cancer

Angiogenesis promotes tumorigenesis and is regulated by cancer cells. A higher tumor microvessel density is always observed in TNBC compared to other BC subtypes (193). Vascular endothelial growth factor (VEGF) is a crucial regulator of angiogenesis in TNBCs (190). For instance, bevacizumab is a monoclonal antibody that induces VEGF blockade. Bevacizumab was initially assessed in metastatic TNBC and displayed good PFS when examined in Phase II studies (194). The rationale for the assessment of antiangiogenic agents against TNBC in neoadjuvant settings includes the fact that cancer invasion and metastasis need neovascularization. Enhanced tumor cell escape from the original tumor is followed by intravasation into the circulation (195, 196). The GeparQuinto clinical Phase III neoadjuvant study reported the improved pCR rates from 33% to 43% with the addition of bevacizumab to anthracycline–taxane combinatorial regimen (197). The UK-led Phase III clinical study (ARTEMIS) also delineated the administration of 4 cycles of bevacizumab to the above combinatorial regimen. The resulting combined pCR rate for both HR+ and HR- types was 22% with bevacizumab and 17% for chemotherapy alone (198). However, bevacizumab did not display substantial clinical efficacy to mitigate distant recurrence, and the agent is not recommended for regular usage in neoadjuvant-treated TNBC. Several other antiangiogenic inhibitors, sunitinib and pazopanib, are currently under clinical investigation as neoadjuvant therapies against TNBCs.

#### 4.4.8 Neoadjuvant Immunotherapies: Successes and Failures

Tumor heterogeneity provides a background for cancer adaptability and failure of several neoadjuvant and adjuvant therapies. Majority of BCs acquire drug resistance through selective enrichment of residual (resistant) tumor cells within the tumor microenvironment (TME) (199, 200). TNBCs are surrounded by divergent TME. Many cytotoxic agents were reported to transform EMT responses, accompanied by the invasion, and metastasis (199). Other agents were shown to reverse EMT and impair metastasis (201, 202).

Neoadjuvant immunotherapies improve the pCR rate with a favorable prognosis, but the prognosis for TNBC patients with residual cancer is variable and less promising in obese individuals (203, 204). The 5-year recurrence rate is predominantly higher for patients with extensive residual disease than that in patients with minimal residual disease after neoadjuvant therapy (205). Additionally, favorable clinical outcomes for continuing systemic therapy in extensive residual TNBC patients were not reported for patients who are at higher recurrence risk despite receiving taxane- and anthracycline-containing neoadjuvant therapy. The tumor-specific ctDNA (circulating DNA) in the plasma of TNBC patients could be used as a marker for the identification of minimal residual disease following neoadjuvant chemotherapy and enables tracking of early therapeutic intervention to clear the ctDNA (142). For instance, the identification of ctDNA in the plasma of these patients would benefit clinicians to choose specific immunotherapy with pembrolizumab. Monitoring of patients with ctDNA for metastasis was recommended (142). Furthermore, the immune-oncology therapeutic strategies are gaining attention as a promising treatment for TNBCs. Application of checkpoint inhibitors with neoadjuvant chemotherapy in the I-SPY2 trials altered the treatment paradigms that promised to attain good clinical outcomes. However, the immune-related adverse toxicities require further testing. There is a need for the development of neoadjuvant therapies against TNBC type 4 subgroups to enhance the maximal clinical response (142).

The failure to attain effective pCR rates with several neoadjuvant therapeutic agents is associated with MDR that is mediated through several cell signaling pathways, including G protein-coupled receptor (GPCR)/RAS/RAF/MAPK, IL6-JAK/STAT3/mTOR, and HER2/PI3K-AKT signaling networks (206). Other signaling pathways that promoted drug-resistant BCs activated the expression of transporter gene ABCG2, survival-linked genes STAT3 and HER2, transcription factors (AP-1 and NF-κB), and epigenetic factors HDACs (207). The raised IL-6 and IL-8 secretion and STAT3 activity are crucial for the development of MDR in BC cells, limiting the pharmacological activity of chemotherapeutic drugs (208–210).

#### 4.4.9 Neoadjuvant Endocrine Immunotherapies and Relevant Clinical Trials

A plethora of clinical reports indicated that neoadjuvant therapy can generate a similar overall and disease-free survival rate observed during adjuvant therapy in ER-BC patients with higher rates of breast-conserving surgery (211–214). ER+ BCs did not demonstrate effective clinical responses after the administration of neoadjuvant therapeutic molecules and hence need the administration of robust alternatives in both normal and obese individuals (213, 214). Among these alternatives, the addition of neoadjuvant endocrine therapy is one of the significant options of chemotherapy to combat TNBCs. Major types of neoadjuvant endocrine therapy include the application of an ER antagonist (tamoxifen), a selective ER modulator (fulvestrant), and aromatase inhibitors (letrozole and anastrozole). All those agents were shown to block estrogen/ER signaling, ER expression, and/or estrogen synthesis (215). These drugs are recommended for patients who are not suitable for surgical interventions. The optimal duration of the therapeutic regimen is hard to define for all patients compared to neoadjuvant chemotherapy alone (215).

Adjuvant endocrine therapy is a combination of endocrine therapy with chemotherapy drugs that have already shown effectiveness in a metastatic situation to overcome BC endocrine resistance (216). This approach provides a good therapeutic perspective for obese patients with TNBCs. After the introduction of everolimus in the treatment of patients with metastatic BC (217), the adjuvant studies were subsequently started (e.g., NCT01674140, NCT01805271 www.clinicaltrial.gov). Everolimus was substantially evaluated as the adjuvant therapy agent in two clinical studies, i.e., SWOG1207 (NCT01674140). The trial is designed to randomly assign everolimus or placebo to high-risk premenopausal and postmenopausal patients who are on their standard adjuvant chemotherapy. Another clinical study (NCT01805271) evaluated the addition of everolimus to adjuvant endocrine therapy in high-risk ER+/HER2- BC patients. The study demonstrated an improved disease-free survival after 1 year of treatment.

Another option is to use a combination of CDK4/6 inhibitors that are less toxic. Dowsett et al. (218) described the effect of palbociclib along with 3 months of neoadjuvant endocrine therapy with letrozole. Results of this study have concluded the substantial palbociclib-mediated antiproliferative effect against BC. The percentage of tumors that underwent a complete cell cycle arrest, indicated in the form of a Ki-67 value <2.7%, during neoadjuvant therapy was reported to be enhanced from 58.5% to 90.4% by the addition of palbociclib (218). Novel effective combination therapies, adjuvant therapy studies at neoadjuvant settings must be conducted for all CDK4/6 inhibitors against TNBCs (219).

Ribociclib is a CDK4/6 inhibitor reported to mitigate recurrence in patients with HR+ early BC when it is preferred in combination with standard adjuvant endocrine therapy (NCT03078751) (220). Ribociclib administration demonstrated an effective clinical efficacy with limited toxicity when combined with ET in patients with HR+, HER2- advanced BC. The purpose of this study was to evaluate the preliminary safety and tolerability of ribociclib to standard adjuvant ET in patients with HR+, HER2- high-risk BCs [https://clinicaltrials.gov]. Application of these molecules is preferred for the treatment of normal-weight and obese BC patients.

#### 4.4.10 Radiation Therapy

Our search identified a total of 36 trials’ clinical data that compared the efficacy of radiotherapy to surgery in BC patients. Majority of the trials indicated that additional radiation therapy can strongly decrease the rate of local recurrence. Radiotherapy after surgery is three times more effective than surgery alone. However, the 10-year survival rate analysis for both methods did not show a significant difference (221). The assessment did not compare the treatment outcomes in obese vs. normal-weight BC patients.

#### 4.4.11 Aromatase Inhibitors

Aromatase is the main enzyme responsible for estrogen biosynthesis in postmenopausal women (222). Aromatase is found in adipose tissue of both normal breast and tumor tissues (223). The microsomal enzyme aromatase is encoded by human CYP19 single-copy gene located on chromosome 15p21.2, which can catalyze the conversion of testosterone and androstenedione to 17-beta-estradiol and estrone (224). Aromatase inhibitors are reported to reduce the circulating estrogen level by blocking the aromatase enzyme activity. However, they are found to be effective only in postmenopausal women. The inhibitors provide time-limited effects, as estrogen levels eventually return to normal once aromatase inhibitors are withdrawn as a BC therapeutic regimen (225). Two types of aromatase inhibitors were designed: steroidal and nonsteroidal agents. Exemestane is a third-generation steroidal aromatase inhibitor and can be administered as an adjuvant therapy in postmenopausal women with early-stage or advanced BCs (226). Everolimus, an immunosuppressant, can be coadministered (combinatorial regimen) with exemestane. This approach greatly enhanced the PFS compared to the exemestane BC monotherapy alone (227).

#### 4.4.12 Application of Estrogen Receptor Antagonists

ERs are activated by estradiol to foster cell differentiation and proliferation in BCs (228). Tamoxifen citrate forces BC to regress, acting as a competitive ER antagonist. The administration of tamoxifen in certain BC patients can reduce the need for oophorectomy, hypophysectomy, or adrenalectomy (229, 230). According to numerous reports, tamoxifen citrate arrests cancer cell growth, induces apoptosis, and modulates the activation of many proliferation- and survival-related signaling proteins (such as calmodulin, protein kinase C, protooncogene c-myc, and transforming growth factor-beta) (231). However, prolonged treatment with tamoxifen results in the development of resistance (232).

#### 4.4.12 Targets for HER2+ Metastatic Breast Cancers

HER2 gene amplification and overexpression were found in approximately 20%–25% of BCs (233). HER2 activates several downstream pathways required for the proliferation of cancer cells (234). Therefore, HER2 inhibitors are used for HER2+ BC treatment. For instance, trastuzumab is produced as a recombinant humanized monoclonal antibody and specifically used against BCs overexpressing HER2 (235). It has been confirmed that trastuzumab improves the clinical benefits when combined with other chemotherapy agents in HER2+ metastatic BCs (236). There are also other novel options including gene therapy, oncogene inactivation, augmentation of tumor suppresser genes, cell-target suicide, chemoprotection approach, virus-mediated oncolysis, and immunomodulation (237), which require intensive clinical testing.

### 4.5 Surgical Interventions

#### 4.5.1 Breast Surgery

This includes mastectomy and breast-conserving surgery that consists of lumpectomy, wide excision, quadrantectomy, and da Vinci robot-assisted axillary lymph node dissection for BC patients with obesity (238). A prospective study was conducted for 20 years of follow-up of patients with radical mastectomy (Halsted). The study compared the efficacy of breast-conserving surgery to other more radical BC surgeries (radical mastectomy) in normal and obese individuals. It has been concluded that breast-conserving surgery is a better treatment choice for patients with relatively small tumors. However, the data indicated that the long-term survival rate does not differ for the compared types of surgeries (239). Primary and secondary lymphedema are the most significant complications among the BC patients who underwent radical mastectomy. During both types of surgical intervention, the time of the surgical procedures should be considered as the impact factor for the outcome in older patients. Two independent population-based studies revealed that the prolonged time of the surgical intervention could be conducive to the low overall and disease-specific survival (240).

#### 4.5.2 Oophorectomy

For less than 50-year-old patients with early BC, the removal of ovaries (241) substantially increased long-term survival in the absence of follow-up chemotherapy. The Early BC Trialists’ Collaborative Group reported 130 deaths and 153 recurrences among 2,102 patients (<50 years old). In this cohort, the 15-year survival rate was highly increased among the patients who underwent ovarian ablation [52.4% vs. 46.1%, 6.3 (SD 2.3)]. The relatively low number of deaths (per 100 women, log rank 2p = 0.001) also correlated with significantly improved recurrence-free survival (45.0% vs. 39.0%, 2p = 0.0007) (242).

### 4.6 Obesity and Complementary Therapeutic Implications Against Breast Cancer

The improvement in surgical techniques and medications can effectively mitigate the mortality rate of BC patients. However, a plethora of scientific evidence reported that we need to apply different complementary approaches to improve the overall survival rate and the quality of life (QoL) of BC survivors with obesity. Therefore, it is crucial to mitigate obesity and its related complications pertaining to the BC incidence and enhancement using complementary nutritional interventions as adjuvant therapy in addition to the conventional therapeutic management.

#### 4.6.1 Physical Activity as a Promising Weight-Reducing Approach in Breast Cancer Patients

Regular endurance exercise is believed to be an effective approach to increase QoL in obese patients who may be prone to BCs. The cardiorespiratory function, physical fitness, and fatigue in BC survivors can be modulated using exercise strategies (243). Aerobic exercise therapy was shown to improve social/family wellbeing, mental health (244), and self-esteem (245) in addition to other physical benefits. More than 150 min of moderate or 75 min of intense aerobic exercise per week and more than 2 days of strength training per week are recommended to complement the adjuvant or hormone-based chemotherapies (American Cancer Society Guidelines) (226–230). Exercise is a safe and feasible technique for improving the physical functioning, fatigue, and overall QoL (246). Additionally, several studies showed that moderate physical activity can increase chemotherapy effectiveness (245). The exercise-based complementary strategy was suggested to improve QoL of obese BC patients. A decrease in serum CRP after intensive physical training has been reported (247). Relevant studies were summarized in **Table 3** and indicate positive outcomes of physical activity in obese BC patients (253).

**Table 3:** Effects of physical exercise on BC treatment outcome.

Study	Materials and Methods	Outcomes	Conclusions
Al Schwartz 2000 (248)	8-week home-based exercise program	Fewer days of high fatigue and more days of low levels of fatigue after chemotherapy	Exercise may decrease cancer treatment-related fatigue
Young-McCaughan S et al., 1991 (249)	42 exercised cancer patients compared with 29 non-exercised cancer patients	Exercised women had a significantly higher QoL (p = 0.03)	Aerobic exercise can be considered in rehabilitation of patients with cancer
McNeely et al., 2010 (250)	Early vs. delayed implementation of postoperative exercise	Implementing early exercise was more effective in the short-term recovery of upper-limb dysfunction after BC treatment	Exercise can improve shoulder range of motion in women with BC
Bedareski et al., 2012 (251)	3 weeks on bicycle ergometer	Significant improvement in aerobic capacity in BC survivors	A 3-week moderate-intensity aerobic training greatly improved the level of aerobic capacity
Milne et al., 2008 (252)	Immediate exercise group vs. delayed exercise group for 12 weeks of supervised aerobic and resistance training in BC patients after completing adjuvant therapy	QoL increased by 20.8 points in the immediate group compared to a decrease by 5.3 points in the delayed group	Exercise soon after completion of BC treatment results in large improvements in QoL

#### 4.6.2 Healthy Weight and Breast Cancer Risk

Regular maintenance of healthy weight is a critical process, and a number of studies over the past decades proved that being overweight or obese at the time of diagnosis indicates a poor prognosis and relatively causes a higher chance of developing primary and secondary lymphedema (254). The data strongly suggest that body weight is closely linked to the overall clinical outcomes in individuals with BC (255). Furthermore, the pooled analysis of several major prospective cohort studies suggested the positive interaction between body size including height, weight, and risk of postmenopausal BC (256). Body weight is also found to be the only risk factor between BC incidence and subsequent prognosis among women in a randomized trial of adjuvant ovarian ablation (257). Furthermore, obesity is also characterized with a higher risk of developing postsurgical lymphedema (258) that is one of the most common complications of BC surgery and radiation therapy (259). Modest weight loss (5%–10%) is recommended for BC women who are overweight or obese (260). A 12-week weight loss diet and training trial revealed a reduction in breast ductal fluid estrogens with weight loss in postmenopausal women (261). There are several interventions that can be employed according to the patient’s needs and diagnostic factors (**Table 4**).

**Table 4:** The basic management strategies for overweight and obese individuals with BCs.

BMI 24.9–30 kg.m^2^	30–40 kg.m^2^	>40 kg.m^2^
Dietary changes (262)	Dietary changes (262)	Dietary changes (262)
Physical activity (263)	Physical activity (263)	Physical activity (263)
	Medications for obesity (264)	Medications for obesity (264)
		Bariatric surgery (265)

#### 4.6.3 Dietary Choices as Complementary Approach for Breast Cancer Treatment

Healthy and balanced diet is an important component of recovery after and during BC treatment. It helps not only to improve QoL but also to provide better treatment outcomes. A diet rich in vegetables, fruits, and whole grains is the preferred regimen for obese women with BC (266). Moreover, a diet with low saturated fats and limited alcohol consumption are beneficial strategies to mitigate BC pathophysiology and adiposity-induced complications. Several pro-oncogenic pathways are targeted by healthy diet components. Previous research reports suggested a 43% decrease in overall mortality in BC survivors who consumed high-vegetable and -whole grain diets (267). Another study indicated a decrease in serum CRP, matrix metalloproteinase 9 (MMP-9), vascular cell adhesion molecule 1 (VCAM), and P-selectin levels in patients on a high-fiber and low-fat diet (268). A low-fat diet reduced the risk of invasive BC, suggesting that a low fat intake has potential long-term benefits (269). Restricted alcohol intake was also shown to be beneficial in several observational studies (270). Excessive ethanol intake can alter hormonal hemostasis and increase BC susceptibility (271). However, the association between some protein-based diets, including soy and dairy intake, and the increased risk of BC remains controversial. Several reports suggested the protective role of dairy products in BC patients (272–275). The evidence of isoflavone-related benefits in BC warrants further investigation (276).

#### 4.6.4 Dietary Supplement as a Complementary Approach in Breast Cancer Patients

There is insufficient scientific evidence available to support the benefits of dietary supplements, including vitamins, minerals, and traditional medicinal herbs. However, BC patients reported the highest intake of dietary supplements (277). Notably, BC patients exhibited higher rates of vitamin D insufficiency (278), although calcium and vitamin D supplements failed to reduce the incidence of invasive BC in postmenopausal women. A recent study reported a comparative efficacy and safety of natural therapies and herbal medicines against invasive BC (279). However, herbal medicine efficacy and health adverse effects remain underaddressed. The application of herbal remedies warrants further investigation, especially in advanced and metastatic BC patients (280). A remedy with established biochemical or clinical benefits in BC patients with nutritional deficiency may be recommended as a supplemental anticancer intervention (281). However, robust clinical trials are required to confirm the efficacy and absence of adverse effects in BC patients who consume herbal medicinal and/or nutritional agents as dietary supplements for weight reduction.

## 5 Conclusions and Summary

Obesity is one of the significant health-related complications in the modern affluent society. Approximately 2 billion adults were considered overweight or obese worldwide in 2015, accounting for 38%–40% of the world’s population (282). Obesity is associated with higher morbidity and mortality rates for many chronic diseases, including BC. The incidence of obesity has been increasing in the aging population (283–286). Excessive adipose tissues and adipocyte hypertrophy were associated with higher levels of local estrogen and chronic inflammation, the factors responsible for BC development and progression in postmenopausal women. However, not all questions related to the role of adipose tissue in cancer patients have been clarified. Moderately overweight patients have shown better survival outcomes after extensive chemotherapy treatments, suggesting that the “obesity paradox” should be thoroughly investigated. BMI has significant implications for BC treatment, the development of novel targeted therapies, and better BC prognosis. Current conventional strategies should be complemented by weight management and primary prevention programs. Early diagnosis and treatment markers may also include some adiposity-related targets. The “personalized medicine” approach should include diet and lifestyle approaches to reach better treatment outcomes. However, overweight reduction, the target for novel therapeutic and preventive measures, should be considered individually after consideration of all confounding factors. Physical activity, diet changes, and optimal weight management should be considered as the most valuable complementary methods that can help to reach better BC surgery and chemotherapy and/or radiotherapy outcomes.

## Data Availability Statement

The original contributions presented in the study are included in the article/supplementary material. Further inquiries can be directed to the corresponding authors.

## Author Contributions

KC, JZ, NB, and OS conceptualized and designed the study. CT, YB, IR, OS, MS, JCZ, XZ, PL, JL, RF, NB, and KC performed the literature analysis and wrote different parts of the original article draft. NB, KC, MS, OS, RF, and PL revised, edited, and extended the final draft. All authors have reviewed and approved the article before submission.

## Funding

The authors are thankful to the National Natural Science Foundation of China (No. 81703158) and the Russian Academic Excellence project “5-100” of the Sechenov University, Moscow, Russia, for the financial support.

## Conflict of Interest

The authors declare that the research was conducted in the absence of any commercial or financial relationships that could be construed as a potential conflict of interest.

## Publisher’s Note

All claims expressed in this article are solely those of the authors and do not necessarily represent those of their affiliated organizations, or those of the publisher, the editors and the reviewers. Any product that may be evaluated in this article, or claim that may be made by its manufacturer, is not guaranteed or endorsed by the publisher.
